# Multimodal brain features at 3 years of age and their relationship with pre-reading measures 1 year later

**DOI:** 10.3389/fnhum.2022.965602

**Published:** 2022-08-22

**Authors:** Kathryn Y. Manning, Jess E. Reynolds, Xiangyu Long, Alberto Llera, Deborah Dewey, Catherine Lebel

**Affiliations:** ^1^Department of Radiology, University of Calgary, Calgary, AB, Canada; ^2^Alberta Children’s Hospital Research Institute, Calgary, AB, Canada; ^3^Hotchkiss Brain Institute, University of Calgary, Calgary, AB, Canada; ^4^Telethon Kids Institute, The University of Western Australia, Perth, WA, Australia; ^5^Centre for Cognitive Neuroimaging, Donders Institute for Brain, Cognition and Behavior, Nijmegen, Netherlands; ^6^Department of Cognitive Neuroscience, Radboud University Medical Centre, Nijmegen, Netherlands; ^7^Department of Pediatrics, University of Calgary, Calgary, AB, Canada; ^8^Community Health Sciences, University of Calgary, Calgary, AB, Canada

**Keywords:** neurodevelopment, MRI, reading, pediatric, diffusion imaging, resting state fMRI, brain, APrON

## Abstract

Pre-reading language skills develop rapidly in early childhood and are related to brain structure and functional architecture in young children prior to formal education. However, the early neurobiological development that supports these skills is not well understood. Here we acquired anatomical, diffusion tensor imaging (DTI) and resting state functional MRI (rs-fMRI) from 35 children at 3.5 years of age. Children were assessed for pre-reading abilities using the NEPSY-II subtests 1 year later (4.5 years). We applied a data-driven linked independent component analysis (ICA) to explore the shared co-variation of gray and white matter measures. Two sources of structural variation at 3.5 years of age demonstrated relationships with Speeded Naming scores at 4.5 years of age. The first imaging component involved volumetric variability in reading-related cortical regions alongside microstructural features of the superior longitudinal fasciculus (SLF). The second component was dominated by cortical volumetric variations within the cerebellum and visual association area. In a subset of children with rs-fMRI data, we evaluated the inter-network functional connectivity of the left-lateralized fronto-parietal language network (FPL) and its relationship with pre-reading measures. Higher functional connectivity between the FPL and the default mode and visual networks at 3.5 years significantly predicted better Phonological Processing scores at 4.5 years. Together, these results suggest that the integration of functional networks, as well as the co-development of white and gray matter brain structures in early childhood, support the emergence of pre-reading measures in preschool children.

## Introduction

Reading is an essential skill that plays a fundamental role in academic achievement, social engagement with the world and peers, and mental health (Dehaene-Lambertz et al., 2006a; Torppa et al., 2006; May et al., 2011; Lonigan et al., 2013). Language skills develop dramatically throughout early childhood and lay the foundation for later reading skills. Phonological awareness and speeded naming in particular are strong predictors of future reading success (Lonigan et al., 2009; Snowling and Hulme, 2011). These skills are supported by neurobiological processes that are primed *in utero* (May et al., 2011) and develop rapidly during early life. A thorough understanding of the early regionally specific structural and functional brain features associated with later reading abilities is essential to determine how reading difficulties emerge, and to help appropriately target early language and reading interventions (Zijlstra et al., 2020).

Reading involves a left lateralized network of white matter connections that support communication between cortical regions (Torppa et al., 2006; Schlaggar and McCandliss, 2007; Zijlstra et al., 2020). Frontal and temporal-parietal cortical regions are connected *via* dorsal white matter pathways including the arcuate fasciculus and superior longitudinal fasciculus (SLF). Frontal, temporal, and occipital regions are connected by ventral white matter pathways [inferior longitudinal (ILF), inferior fronto-occipital (IFO), and uncinate fasciculi (UF)] (Klingberg et al., 2000; Brown et al., 2001; Hickok and Poeppel, 2004; Welcome et al., 2011). Throughout childhood, both cortical structure (Szaflarski et al., 2006; Holland et al., 2007) and brain functional patterns (Dehaene-Lambertz et al., 2002, 2006b; Peña et al., 2003) become more left-lateralized. White matter structures, such as the arcuate fasciculus, also demonstrate left lateralization in early life (O’Muircheartaigh et al., 2013; Dubois et al., 2016), and are linked to language skill development in young children (Reynolds et al., 2019b).

Relationships between reading performance and cortical brain structure and development have been observed in adults (Klingberg et al., 2000; Brown et al., 2001; Welcome et al., 2011), adolescents (Kronbichler et al., 2008; Lebel et al., 2013), and school-aged children (Beaulieu et al., 2005; Deutsch et al., 2005; Eckert et al., 2005; Niogi and McCandliss, 2006; Lebel and Beaulieu, 2009). Cortical structure in children and adolescents is associated with phonological processing skills (Lu et al., 2007), with higher baseline reading skills associated with faster changes in gray matter volume in typically developing children aged 5–15 years (Houston et al., 2014; Linkersdörfer et al., 2014). Furthermore, children aged 6–7 years who subsequently received a diagnosis of dyslexia had reduced cortical thickness in left hemisphere reading-related regions compared to children who did not eventually receive a diagnosis (Clark et al., 2014). There is evidence that these alternate development patterns emerge even earlier, with slower proportional growth of left cortical regions was observed in preschool aged children with a family history of reading disorders (Ostertag et al., 2021).

White matter microstructural properties like myelin and axonal packing (Geeraert et al., 2020) support pre-reading skills in preschool children (Walton et al., 2018), and reading skills in adolescents (Ben-Shachar et al., 2007) and adults (Welcome and Joanisse, 2014). Longitudinal studies have also demonstrated that white matter development supports reading gains. Faster changes in white matter fractional anisotropy (FA) and mean diffusivity (MD), that represent faster maturation, are associated with larger gains in reading skills in children with and without developmental disorders (Yeatman et al., 2012a; Treit et al., 2013; Wang et al., 2017). Furthermore, similar changes in white matter microstructure have been demonstrated following intensive reading interventions (Keller and Just, 2009; Huber et al., 2018). While these studies suggest associations between early white matter maturation and reading development in children, it is unclear what early white matter characteristics support pre-reading skills later in childhood. Emerging evidence suggests that arcuate fasciculus and corticospinal tract microstructural properties even in infancy can predict phonological processing and vocabulary at 5 years of age (Zuk et al., 2021). However, the coinciding cortical structures and white matter neurobiological properties that support pre-reading skills in preschool aged children, before formal reading education, are not well-understood.

Activation and functional connectivity with reading related regions are related to language skill gains (Xiao et al., 2016), later reading outcomes (Jasińska et al., 2020), and brain development during childhood (Saygin et al., 2016). The process of learning to read is supported by refined functional architecture (Dehaene et al., 2010; Cross et al., 2021). In general, children with higher reading proficiency show faster development of reading-related brain regions than children with poor reading skills (Yeatman et al., 2012b; Wang et al., 2017; Lebel et al., 2019; Reynolds et al., 2019b). A recent cross-sectional study (Benischek et al., 2020) identified higher functional connectivity within the reading network (Wernicke’s and Broca’s temporal-parietal areas), but more negative connectivity between reading areas and the default mode network (DMN), in young children with better pre-reading skills. The brain’s functional architecture during the preschool period has also been shown to relate to pre-reading skills (Raschle et al., 2012; Saygin et al., 2013; Walton et al., 2018; Reynolds et al., 2019b). Furthermore, pre-readers with a family history of dyslexia show reduced temporal-parietal and temporal-occipital activation during sound matching compared to pre-readers without a family history of dyslexia (Raschle et al., 2012).

White matter structure, cortical volume, and functional connectivity have all been studied separately with respect to reading and pre-reading, but their development is inherently linked. In this study, we aimed to determine how early childhood brain morphometry, white matter microstructure, and functional brain network communication predict measures of pre-reading skills later in childhood. Investigating all measures simultaneously can be challenging, as it increases the number of comparisons and typical univariate analyses do not model the shared and spatially specific development between features. Our data-driven linked independent component analysis (ICA) technique (Llera et al., 2019) combined multiple parameters simultaneously to measure co-variation of white matter and cortical structures across participants as they relate to pre-reading measures. This approach has been used to study neurobiological development and aging as well as childhood disorders like autism spectrum disorder and ADHD (Itahashi et al., 2015; Wolfers et al., 2017). In a subset of participants with high-quality functional MRI data, we quantified how the fronto-parietal language network (FPL) communicates with other networks in the brain that may play a role in pre-reading ability. These included the cerebellar (Alvarez and Fiez, 2018), visual, and DMNs which support memory, visual function, and attention while reading (Smallwood et al., 2013), respectively. We hypothesized that multimodal brain structural properties and inter-network connectivity at 3 years of age would relate to pre-reading measures assessed 1 year later.

## Materials and methods

### Participants

The University of Calgary Conjoint Health Research Ethics Board (CHREB) approved this study (REB13-0020). Informed written consent was obtained from each participant’s legal guardian prior to the commencement of the study, and ongoing verbal assent was obtained from the participants. Participants were 35 children (19 boys/16 girls) selected from the ongoing Calgary Preschool MRI Study (Reynolds et al., 2020) based on the criteria of having high-quality T1-weighted and diffusion weighted scans at 3.5 years (3.49 ± 0.14; range 3.25–3.75 years) and a pre-reading assessment at age 4.5 years (4.50 ± 0.16; range 4.25–4.75 years). The University of Calgary CHREB approved this study (REB13-0020). Informed written consent was obtained from each participant’s legal guardian prior to the commencement of the study, and ongoing verbal assent was obtained from the participants.

### Language assessments

Children’s pre-reading skills were assessed using the NEPSY-II Speeded Naming and Phonological Processing subtests (∼20 min) at 3.5 and 4.5 years of age. The Speeded Naming subtest assesses rapid semantic access to and production of names of colors and shapes, and the Phonological Processing subtest assesses phonemic awareness (Korkman et al., 2007). Age standardized Speeded Naming Combined Scaled Scores (accounts for both speed and accuracy) and Phonological Processing Scaled Scores were calculated and used in the analysis. On these measures, higher scores are indicative of better performance.

### Magnetic resonance imaging acquisition

All imaging was conducted using the same General Electric 3T MR750w system and a 32-channel head coil (GE, Waukesha, WI) at the Alberta Children’s Hospital in Calgary, Canada. Children were scanned either while awake and watching a movie of their choice, or while sleeping without sedation. fMRI scans during which the child was asleep were excluded from analyses. This approach has been used in our prior work and is effective for scanning young children while awake (Long et al., 2017). Prior to scanning, parents were provided with detailed information on MRI procedures and given the option to complete a practice MRI session in a training scanner to familiarize the child with the scanning environment, or to make use of a take home pack with this information (e.g., noise recordings; Thieba et al., 2018). Families were also provided with a book that incorporates our scanning procedures into an engaging story (Frayne, 2015) and we encouraged the parents/guardians to review the materials with the child.

T1-weighted images were acquired using a FSPGR BRAVO sequence, 210 axial slices; 0.9 × 0.9 × 0.9 mm resolution, TR = 8.23 ms, TE = 3.76 ms, flip angle = 12°, matrix size = 512 × 512, inversion time = 540 ms. Whole-brain diffusion weighted images were acquired using single shot spin echo echo-planar imaging sequence: 1.6 × 1.6 × 2.2 mm resolution (resampled on scanner to 0.78 × 0.78 × 2.2 mm), full brain coverage, TR = 6,750 ms; TE = 79 ms (set to minimum for first year), 30 gradient encoding directions at b = 750 s/mm^2^, and five interleaved images without gradient encoding at b = 0 s/mm^2^ for a total acquisition time of approximately 4 min. Passive viewing fMRI data were acquired while children were watching a movie of their choice, with a gradient-echo echo-planar imaging (EPI) sequence: total sequence time = 8 min and 10 s, 36 axial slices, 3.59 × 3.59 × 3.6 mm resolution, TR = 2,000 ms, TE = 30 ms, flip angle = 60°, matrix size = 64 × 64, 250 volumes.

### Anatomical imaging

Voxel-based morphometry (VBM) processing was undertaken on the T1-weighted images using FSL [FMRIB (Functional Magnetic Resonance Imaging of the Brain) Software Library freely available at fsl.fmrib.ox.ac.uk] to create gray matter maps. N4-bias correction (Tustison et al., 2010) was performed using ANTs (Avants et al., 2009), then images were transformed to radiological orientation. Brain extraction was undertaken using Brain Extraction Tool (BET) *via* the standard FSL VBM protocol; when brain extraction using the default settings was unsuccessful (*n* = 7), BET was performed manually to achieve the best results. Next, images were segmented into gray matter, white matter and CSF. The gray matter images were then affine registered to the NIHPD asymmetrical pediatric brain template (4.5–8.5 years template; this template was used to be consistent with our prior work in the larger Calgary Preschool MRI sample and to permit future analysis spanning 2–8 years) in Montreal Neurological Institute (MNI) standard space (Fonov et al., 2011), resampled to 2 mm isotropic voxels, concatenated and averaged. Using the standard FSL pipeline, these average gray matter images were used to create a study-specific non-linear gray matter template (2 mm isotropic voxels), following which gray matter images were non-linearly registered to the study-specific template, modulated, smoothed (2 mm), and concatenated into one 4D file.

### Diffusion imaging

Raw diffusion images were visually quality checked and all motion-corrupted volumes, or volumes with artifacts were removed prior to processing. Datasets passed final quality assurance checks (*n* = 35) if they had at least 18 high quality diffusion weighted volumes, and two high quality b0 volumes remaining following volume removal. Data was then pipelined through ExploreDTI V4.8.6 (Leemans et al., 2009) to correct for signal drift, Gibbs ringing (non-DWIs), subject motion, and eddy current distortions. FA, axial (AD), and radial diffusivity (RD) image maps were extracted. All measures were included to understand the specific white matter microstructural properties that contribute to pre-reading development. For images where brain extraction during diffusion tensor imaging (DTI) preprocessing did not remove all non-brain material, FSL BET was run on the extracted FA map, and the resulting binary mask was used to mask the FA, AD, and RD images. These DTI maps were then non-linearly warped using ANTs (Avants et al., 2009) to the NIHPD asymmetrical pediatric brain template (ages 4.5–8.5 years) in MNI standard space (Fonov et al., 2011). All of the registered diffusion data was then merged into one four-dimensional image to create a mean FA mask and image for all subjects. The mean FA image was skeletonized with a threshold of FA > 0.2 to create a mean FA skeleton mask. All participants’ FA, AD, and RD images were non-linearly projected onto that skeleton. Analysis was conducted on skeletonized maps of diffusion measures within tracts that have been associated with reading in children (Wandell and Yeatman, 2013): the bilateral uncinate fasciculus (UF), inferior longitudinal fasciculus (ILF), IFO, and SLF. Tracts were identified using the JHU white matter tractography atlas.

### Linked independent component analysis

**FIGURE 1:**
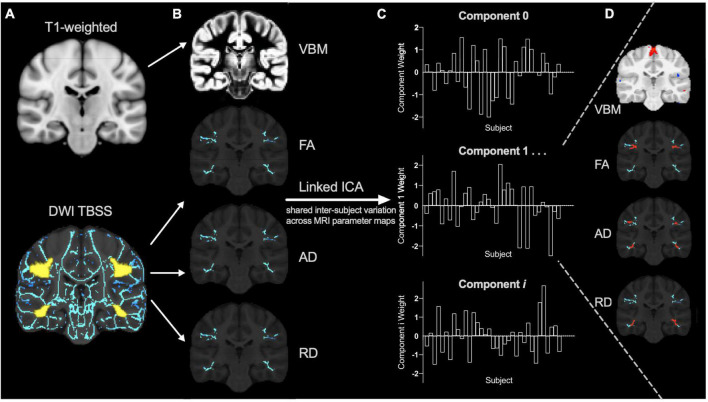
Linked independent component analysis pipeline. **(A)** Preprocessed T1 and diffusion weighted images (DWI) were processed using voxel-based morphometry (VBM) and tract-based spatial statistics (TBSS) pipelines (with the reading-related tracts mask shown in yellow) to create **(B)** individual VBM, fractional anisotropy (FA), axial diffusivity (AD), and radial diffusivity (RD) spatial maps for each subject as input for the linked independent component analysis (ICA). The data is decomposed into a series of linked components comprised of **(C)** component weights that reflect the inter-subject variation shared across **(D)** all input measures (VBM, FA, AD, and RD) in specific brain regions (for example, shown in red).

Gray matter morphometry and skeletonized FA, AD and RD maps were aligned to the same space, concatenated, and then used as input for a linked ICA using FSL tools (Llera et al., 2019). This is a data-driven approach that uncovers the neurobiological variations across multiple MRI parameter maps (Groves et al., 2011) so by including multiple diffusion measures we aim to explain the neurobiology more specifically. Briefly, the concatenated imaging data from all subjects are decomposed into a series of linked independent components. Linked components involve a spatial map for each MRI parameter (Figure 1D) that displays where that combination of MRI metrics co-vary across participants according to a common component weighting (Figure 1C). Each MRI parameter has a relative modality loading in each component (Groves et al., 2012) that contributes a fraction of the total variance explained by that component. The component weight describes the combined relative variability of multiple MRI metrics across participants and decreases the number of multiple comparisons while enabling examination of multiple parameters in conjunction (Figure 1). Functional data could not be incorporated in our linked models because only a subset of participants’ functional data passed quality check procedures.

### Resting state functional magnetic resonance imaging

Resting state functional MRI preprocessing is described in detail elsewhere (Long et al., 2019). Briefly, processing was undertaken using FSL (slice timing, head motion correction, T1 image segmentation, head motion outlier detection, co-registration, and spatial normalization and smoothing) (Jenkinson et al., 2002) and AFNI v17.3.03 (regression of the nuisance signals, band-pass filtering and linear trend removal) (Cox, 1996). fMRI data was registered to a pediatric template in MNI standard space (Fonov et al., 2011). Participants with fMRI data that had < 4 min of low-motion data, or excessive motion (relative frame-wise displacement < 0.3 mm) at any time (Satterthwaite et al., 2013) were excluded from the analysis, resulting in a total of 21 fMRI datasets.

Clean fMRI data (*n* = 21) was temporally concatenated and analyzed using ICA to decompose the data into 20 components or networks. This model order was chosen to adequately model known neural networks, physiological signals, and noise (Wang and Li, 2015). To identify resting state networks (RSNs), the components were compared (using cross correlation) to the 10 most common RSNs (Smith et al., 2009). We focused on the left-lateralized FPL network because it involves regions, including Broca’s and Wernicke’s areas, that are functionally active during cognitive-language tasks (Smith et al., 2009; Reynolds et al., 2019b). Dual regression algorithms were used to back-reconstruct subject-specific RSNs composed of data-driven voxel-wise clusters. Reading is a complex skill that involves communication between distinct networks in the brain that are involved with visual, memory, attention, and language processing. Therefore, we focused on how the FPL network communicates with other relevant brain networks, as recent findings in preschool aged children suggest that both within-network communication as well as integration with other networks support pre-reading skills (Benischek et al., 2020). We investigated the inter-network communication between the FPL and networks responsible for visual processing, memory, and attention. The average timeseries from each of these networks (FPL, visual, DMN, and the cerebellar RSN) was extracted. Inter-network functional connectivity between the FPL network and the three RSNs were calculated for each participant.

### Statistical analysis

This exploratory study used partial correlations controlling for sex. We performed bootstrapping in SPSS (version 26) with 1,000 samples to determine confidence interval of any relationships between the linked ICA component weights at 3.5 years and pre-reading measures at 4.5 years. Sex was included as a covariate because of known sex differences in brain development in this age range (Reynolds et al., 2019a). The inter-network functional connectivity between the FPL and the three RSNs was also tested for correlations with pre-reading measures 1 year later using a partial correlation analysis controlling for sex and age at the time of the scan. As this was an exploratory study, the nominal *p*-values for the relationships between component weights, inter-network functional connectivity and pre-reading measure are reported. We also report if these relationships survive false-discovery rate (FDR) correction for the linked ICA and functional RSNs analyses separately.

## Results

### Pre-reading measures

At 4.5-years, the mean Phonological Processing standard score was 11.9 ± 2.1 and ranged from 7 to 16. The mean Speeded Naming standard score was 12.4 ± 2.3 and ranged from 7 to 18. Speeded Naming and Phonological Processing scores at 3.5 years of age were not correlated with scores obtained at 4.5 years (Speeded Naming: *r* = 0.02, *p* = 0.91; Phonological Processing: *r* = 0.26, *p* = 0.14).

### Linked brain structure features that relate to pre-reading

The masked (bilateral UF, IFO/ILF, and SLF) and skeletonized DTI maps (FA, AD, RD) and the gray matter morphometry maps were input into the linked ICA. The linked ICA generated 14 components. Of these, three components were driven by an individual subject; therefore, these components were not included in the correlation analysis that investigated the relationships between the brain imaging component weights and pre-reading measures. Of the remaining 11 components, two correlated with pre-reading measures. To confirm the validity of components of interest, linked independent component models with 13 and 15 components (model order) were also assessed. The two components described below were reproducible using all three model orders (*p* < 0.05). Intracranial volume was not related to either reading score (*p* > 0.05).

**FIGURE 2:**
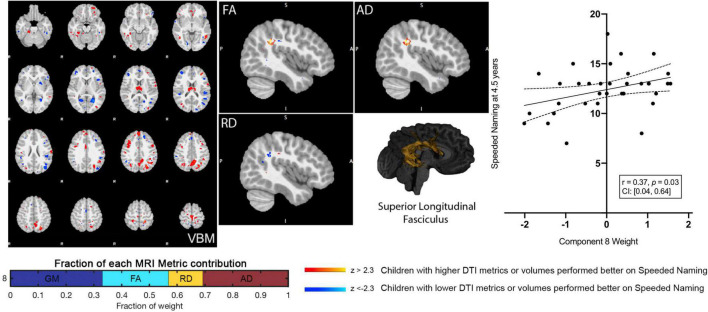
Component 8 was approximately evenly weighed by the voxel-based morphometry (VBM) gray matter, fractional anisotropy (FA), radial diffusivity (RD), and axial diffusivity (AD) contributions. This component was positively correlated with Speeded Naming at 4.5 years. For regions in red (positive clusters), children with larger volumes/higher DTI metrics performed better on Speeded Naming, and for regions in blue (negative clusters), children with smaller volumes/DTI metrics performed better on Speeded Naming. For reference, the superior longitudinal fasciculus is also shown.

Component 8 weights were positively associated with Speeded Naming scores (*r* = 0.37, *p* = 0.03, CI: [0.04, 0.64]) at age 4.5 years (Figure 2), but this relationship did not survive FDR correction (corrected *p* = 0.3). This component involved volumetric variability in the angular gyrus, thalamus, fusiform gyrus, middle temporal regions, and the dorsolateral prefrontal cortex (Table 1) and was linked with bilateral changes in FA and AD along the SLF. Specifically, relatively higher component weights amongst participants represent larger volumes in the sensorimotor regions, the angular gyrus, thalamus, fusiform gyrus, and middle temporal area, smaller volumes in the posterior cingulate and visual association areas and the prefrontal cortex, combined with higher FA and AD, and lower RD (though less pronounced than FA and AD) in the bilateral SLF were related to higher pre-reading scores.

**TABLE 1:** Characterization of component 8 voxel-based morphometry gray matter clusters.

# Pos. voxels	Peak (x, y, z) coordinates	Regions within the cluster	Region labels	Peak z-statistic
1,062	(–21, –45, 61)	BA7, BA39 (L)^†^	Angular gyrus (Wernicke’s area)	5.9
357	(9, –13, 17)	Thalamus		3.83
317	(1, –23, 71)	BA6	Premotor and supplementary motor cortex	4.22
288	(35, 23, 37)	BA8 (R), BA6 (R)	Frontal eye fields, motor association cortex	4.21
184	(35, –37, –15)	Fusiform (R)		3.35
180	(–1, 49, 33)	BA9	Dorsolateral and medial prefrontal	5.17
158	(–35, –57, 39)	BA39 (L)	Angular gyrus (Wernicke’s area)	5.22
141	(57, –35, –15)	BA21 (R)	Middle temporal gyrus	5.01
137	(–51, –51, –3)	Fusiform (L)		6.18
132	(27, –61, 43)	BA39 (R)	Angular gyrus (Wernicke’s area)	4.31
75	(–65, –17, 25)	Primary sensory (L)		3.79
74	(25, –41, 63)	BA5 (R)	Somatosensory association cortex	3.51
72	(–37, 41, 19)	BA10 (L), BA46 (L)	Anterior and dorsolateral prefrontal cortex	4.77
66	(–59, 3, 17)	BA44 (L)	Broca’s area pars opercularis	3.84
63	(–3, 27, –7)	BA32 (L)	Dorsal anterior cingulate cortex	3.23
60	(–51, 1, 41)	BA6 (L)	Premotor and supplementary motor cortex	3.09
56	(3, 1, 33)	BA24 (R)	Ventral anterior cingulate cortex	3.2
54	(–43, –45, 41)	BA40 (L)	Supramarginal gyrus (Wernicke’s area)	4.61

**# Neg. voxels**	**Peak (x, y, z) coordinates**	**Regions within the cluster**		**Peak z-statistic**

308	(–25, –59, 7)	BA23 (L), BA18 (L), BA30 (L)	Ventral posterior cingulate cortex, secondary visual cortex	5.73
290	(–47, –51, 17)	BA39 (L)	Angular gyrus (Wernicke’s area)	5.61
255	(–39, –77, 27)	BA19 (L), BA39 (L)	Visual association, angular gyrus (Wernicke’s area)	4.17
215	(35, 47, 5)	BA10 (R)	Anterior prefrontal cortex	4.2
119	(19, –59, 7)	BA23 (R), BA18 (R)	Ventral posterior cingulate cortex, secondary visual cortex	4.01
80	(47, –35, 21)	BA22 (R), BA40 (R)	Superior temporal and supramarginal gyrus (Wernicke’s area)	3.89
62	(1, 33, 41)	BA8 (R), BA8 (L)	Frontal eye fields	3.63
61	(–23, 7, 1)	Putamen (L)		3.33
59	(–21, –93, 21)	BA18 (L)	Secondary visual cortex	3.98
55	(–45, 21, 7)	BA45 (L)	Broca’s area (pars triangularis)	3.75
54	(–49, –37, –19)	BA20 (L)	Inferior temporal gyrus	4.06

^†^BA, Brodmann area; L, left; R, right.

**FIGURE 3:**
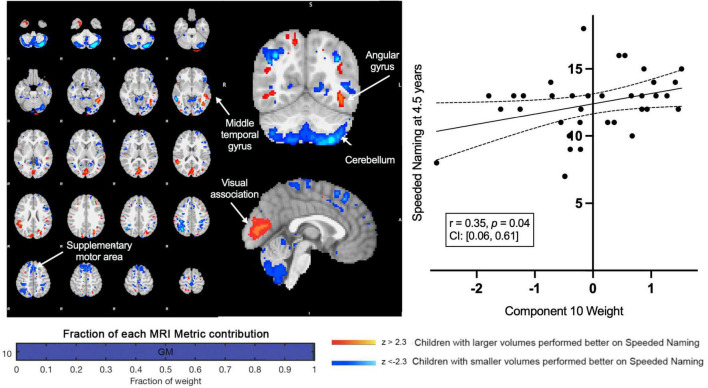
Component 10 was dominated by gray matter contributions. This component was positively correlated with Speeded Naming performance at 4.5 years. For regions in red (positive clusters), children with larger volumes performed better on Speeded Naming, and for regions in blue (negative clusters), children with smaller volumes performed better on Speeded Naming.

A second component (Component 10) was dominated by volumetric variability in the cerebellum, precuneus, angular gyri, and areas in the occipital cortex (visual regions), and was positively associated with Speeded Naming (*r* = 0.35, *p* = 0.04, CI: [0.06, 0.61]) at age 4.5 years (Figure 3). This association did not survive FDR correction (corrected *p* = 0.2). Smaller cortical volumes in the cerebellum, and supramarginal and angular gyri, and relatively larger volumes in visual areas in the occipital lobe and the fusiform in participants at 3.5 years (Table 2) were associated with higher pre-reading scores at 4.5 years.

**TABLE 2:** Characterization of component 10 voxel-based morphometry gray matter clusters.

# Neg. voxels	Peak (x, y, z) coordinates	Regions within the cluster	Region labels	Peak z-statistic
9,016	(–29, –77, –41)	Cerebellum (L)^†^		11.8
1,670	(13, 31, 59)	BA8 (R)	Lateral and medial supplementary motor area	5.64
1,186	(35, –65, 41)	BA39 (R)	Angular gyrus (Wernicke’s area)	8.28
669	(–47, 33, –5)	BA47 (L)	Fusiform gyrus	5.05
486	(–37, –37, 45)	BA40 (L)	Supramarginal gyrus (Wernicke’s area)	6.91
398	(–3, –19, 75)	BA6 (L)	Premotor and supplementary motor cortex	4.53
245	(–15, 53, 29)	BA9 (L)	Dorsolateral prefrontal cortex	4.1
234	(59, –41, –5)	BA21 (R)	Middle temporal gyrus	6.91
167	(–63, –3, –7)	BA22 (L)	Superior temporal gyrus (Wernicke’s area)	3.76
111	(–49, –59, 7)	BA39 (L)	Angular gyrus (Wernicke’s area)	4.49
90	(49, 1, 13)	BA6 (R)	Premotor and supplementary motor cortex	5
88	(41, –37, –19)	Fusiform (R)		4.41
78	(–13, –89, 33)	BA19 (L)	Visual association	4.31
76	(15, 7, –19)	BA11 (R)	Orbitofrontal cortex	3.62
73	(19, –71, 47)	BA7 (R)	Somatosensory association cortex	3.93
69	(–42, –6, 45)	BA6 (L)	Premotor and supplementary motor cortex	4.91
66	(21, –37, 61)	BA5 (R)	Somatosensory association cortex	3.69
64	(19, –9, 61)	BA6 (R)	Premotor and supplementary motor cortex	5.51
63	(–43, –34, –12)	BA20 (L)	Inferior temporal gyrus	3.85
61	(29, 23, 41)	BA8 (R)	Frontal eye fields	4.49
60	(15, –77, –5)	BA18 (R)	Secondary visual cortex	3.71
58	(–23, 13, –21)	BA47 (L)	Inferior frontal gyrus (pars orbitalis)	4.1
58	(–49, 43, –15)	BA47 (L)	Inferior frontal gyrus (pars orbitalis)	3.53
55	(29, –11, 69)	BA6 (R)	Premotor and supplementary motor cortex	4.01
51	(21, –95, –5)	BA18 (R)	Secondary visual cortex	4.89

**# Pos. voxels**	**Peak (x, y, z) coordinates**	**Regions within the cluster**		**Peak z-statistic**

1,238	(15, –87, 27)	BA19 (R)	Visual association	5.61
451	(–45, –49, –15)	Fusiform (L)		5.95
444	(51, –47, 23)	BA39 (R)	Angular gyrus (Wernicke’s area)	5.67
247	(–29, –69, 33)	BA39 (L)	Angular gyrus (Wernicke’s area)	5.54
243	(–51, –31, –7)	BA21 (L)	Middle temporal gyrus	5.12
195	(23, 5, –41)	BA36 (R)	Perirhinal cortex	4.47
101	(–31, –49, –5)	BA19 (L)	Visual association	4.75
87	(–43, –71, 9)	BA19 (L)	Visual association	4.96
83	(45, –55, –3)	Fusiform (R)		3.88
67	(15, –55, 57)	BA7 (R)	Somatosensory association cortex	5.83

^†^BA, Brodmann area; L, left; R, right.

### Resting state networks and pre-reading

**FIGURE 4:**
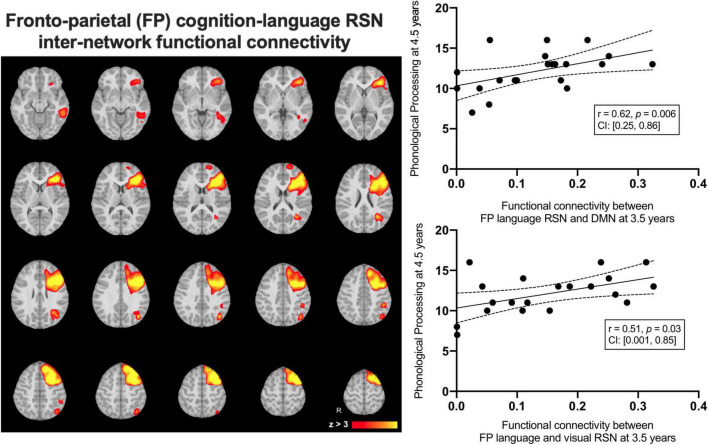
The group average left-lateralized fronto-parietal resting state network (left) and the linear relationships between inter-network functional connectivity at 3.5 years predicting Phonological Processing scores at 4.5 years of age.

We examined average inter-network functional connectivity between the FPL RSN (Figure 4) and three other networks including the DMN, the occipital pole visual and the cerebellar RSNs. The inter-network functional connectivity (correlation between average RSN time series) between the FPL and the DMN assessed at 3.5 years of age was correlated with Phonological Processing scores 1year later at 4.5 years of age (*r* = 0.62, *p* = 0.006, CI: [0.25, 0.86]) and this relationship survived FDR correction (corrected *p* = 0.02). Inter-network functional connectivity between the occipital pole visual RSN and the FPL at 3.5 years of age was associated with Phonological Processing scores one year later (*r* = 0.51, *p* = 0.03, CI: [0.001, 0.85]), but this did not survive FDR correction (corrected *p* = 0.05). Inter-network functional connectivity was not significantly related to either structural linked component weights.

## Discussion

In this exploratory study of typically developing young children, we identified multiparametric brain imaging features at 3.5 years of age that were associated with pre-reading measures 1 year later. Variations in brain volume (angular gyrus, lingual gyrus, thalamus, fusiform gyrus, middle temporal regions, cerebellum, precuneus, and the dorsolateral prefrontal cortex), SLF microstructure, and inter-network functional connectivity of the FPL network at 3.5 years were associated with better pre-reading skills at 4.5 years. More mature SLF microstructure coincided with cortical morphometry variations in portions of Wernicke’s area and Broca’s area. Both are considered critical language comprehension and production regions and were related to better pre-reading scores 1 year later. Also, higher functional connectivity between the FPL and the DMN and visual networks significantly predicted better pre-reading measures 1 year later. Together these findings suggest that gray and white matter structure, as well as inter-network functional communication, contribute to the development of pre-reading skills in young children.

Our multimodal approach was able to capture co-occurring neurobiological variations (Wolfers et al., 2017; Llera et al., 2019) in gray and white matter structures. Shared inter-subject variation (Component 8) of gray matter volume in reading-related regions (e.g., supramarginal and angular gyrus and dorsolateral prefrontal cortex) co-varied with microstructural measures (FA, AD, and a smaller contribution of RD) of the white matter that connects them (SLF). Reading-related brain areas such as the SLF and the cortical regions identified continue to develop throughout childhood and even into adulthood (Lebel et al., 2008) as the brain refines and integrates inter-network communications. The SLF is a key dorsal pathway involved in auditory-motor integration and the phonological aspects of both reading and speech (Vigneau et al., 2006). The associations between the SLF and pre-reading measures is consistent with prior longitudinal diffusion MRI research that has reported relationships between arcuate fasciculus (a component of the SLF) microstructure and reading skills in older children (Wandell and Yeatman, 2013). Furthermore, arcuate fasciculus microstructure during infancy is associated with phonological awareness skills and vocabulary knowledge in kindergarten (Zuk et al., 2021). Our findings are also consistent with prior research that has reported cross-section and longitudinal associations between cortical thickness and volume in these areas and reading abilities in older children. Higher FA and lower MD suggest that participants with increased myelin and/or more tightly packed axons (Beaulieu, 2002; Song et al., 2002, 2003). Because FA generally increases and MD decreases during typical development (Reynolds et al., 2019b), the profiles observed here suggest a more mature SLF in children with better pre-reading skills at age 4.5 years. Congruent cerebral structural development involves both thinning and thickening of the cortex depending on the region and stage of development (Remer et al., 2017). These results suggest that co-occurring white and gray matter structure in early childhood may lay the foundation for more advanced reading abilities throughout childhood and adolescence.

The second component associated with later pre-reading performance was dominated by gray matter volume variations. Smaller volumes in the cerebellum, frontal and sensory regions, and larger angular and visual gyri cortical volumes at 3.5 years of age were related to better Speeded Naming scores 1 year later. The cerebellum plays a critical role in reading development through both the dorsal and ventral circuits that support phonological and semantic processes (Alvarez and Fiez, 2018; Benischek et al., 2020). The inclusion of dorsal and ventral cerebellar gray matter regions in this component suggests that the cerebro-cerebellar pathway plays a key role in the development of pre-reading skills. The angular gyrus also plays a role in language and reading, including orthographic processing, with increased functional interactions with Broca’s and Wernicke’s areas and the visual word form area during reading (Segal and Petrides, 2013; Benischek et al., 2020). Better language ability is related to increased activation in this region (Van Ettinger-Veenstra et al., 2016), and disruptions to functional connectivity with temporal and occipital language regions have been reported in adults with dyslexia compared to controls (Horwitz et al., 1997). The visual system is also important for reading, with sensitivity of the visual cortex for word visibility increasing throughout childhood (Ben-Shachar et al., 2011). Connectivity differences between visual areas and other language and reading regions have been observed in poor compared to good readers (Wandell et al., 2012) suggesting that earlier refinement of the cerebellum and somatosensory cortical regions, combined with growth of the visual cortex, relate to better pre-reading skills in the preschool period.

Higher inter-network functional connectivity between the left-lateralized FPL network and both the DMN (including bilateral angular gyri) and visual RSNs at 3.5 years of age predicted better Phonological Processing scores 1 year later. Prior functional connectivity studies of reading have reported mixed results, but have generally found increased activation and connectivity amongst reading-related, sensory and motor regions (Weiss-Croft and Baldeweg, 2015). However, across development regions that are typically active during reading tasks do not exclusively communicate amongst each other, but instead tend to integrate with other networks including the DMN (Smallwood et al., 2013; Vogel et al., 2013). Our results reflect a similar pattern at a much earlier stage of development, where higher functional connectivity between the FPL and DMN was significantly predictive of Phonological Processing scores in early childhood. This suggests that even before formal reading education, better integration of functional networks support pre-reading ability and may lay the foundation for future reading capability as well.

In most individuals, language and reading are left-lateralized in the brain, with resting state functional connectivity patterns demonstrating increased lateralization throughout early development (Reynolds et al., 2019b; Benischek et al., 2020). Functional connectivity lateralization have been shown to be related to the development of language skills task-based fMRI studies (Szaflarski et al., 2006; Holland et al., 2007; Perani et al., 2011; Yamada et al., 2011; Xiao et al., 2016), where lateralization patterns tend to increase in the dominant hemisphere until a plateau in early adulthood that then gradually decreases in laterality over the life span (Szaflarski et al., 2006). The structural brain properties associated with better speeded naming scores were bilateral in our study, which may reflect that while lateralization strengthens over time, a more broad network of regions support pre-reading skills at this early stage of development (Qiu et al., 2011). Our findings suggest that more inter-connected functional network architecture at this stage of development may lay the foundation for later reading abilities, which require seamless communication between brain networks. Functional brain signals are shared among specific cortical regions and networks, and maturation and refinement of the underlying microstructure of pathways connecting those areas may directly support these functional pathways. Children who utilize those particular pathways through frequent exposure to language and reading in early childhood may establish both the structural and functional brain foundations to support reading abilities later in life.

### Limitations

This longitudinal study involved a group of typically developing preschool children with average to high pre-reading skills. Future studies including children with lower scores may assist in determining whether similar patterns hold in children more likely to develop reading problems. We had a tight age range in this study, but the sample size in this exploratory study was relatively small and the statistical tests were under-powered. While VBM has shown similar volumetric results compared to manual region of interest (Asami et al., 2012), other factors like gyrification, surface area and cortical thickness can influence results. Functional connectivity between the FPL and DMN significantly predicted pre-reading measures, specifically phonological processing; however, the structural brain measures associated with pre-reading did not survive a statistical correction. Further studies with larger sample sizes are necessary to confirm these relationships between early brain measures and their influence on a child’s pre-reading abilities later in life.

## Conclusion

In this study we found that linked development of brain white and gray matter structure in early childhood was associated with pre-reading measures 1 year later. In particular, the SLF and the cortical regions that it connects, as well as cerebellar-cerebral reading-related circuits, appear to be important for the development of children’s pre-reading skills. Furthermore, a more functionally integrated FPL network predicted better pre-reading skills 1 year later. This analysis approach demonstrates that co-development of white and gray matter brain structures in early life, as well as the integration of functional networks before formal reading education, are associated with pre-reading abilities in preschool children.

## Data availability statement

The datasets presented in this study can be found in online repositories. The names of the repository/repositories and accession number(s) can be found below: The datasets analyzed for this study can be found in the Calgary Preschool MRI study available through the Open Science Framework (https://osf.io/axz5r/).

## Ethics statement

The studies involving human participants were reviewed and approved by the University of Calgary Conjoint Health Research Ethics Board (CHREB). Written informed consent to participate in this study was provided by the participants’ legal guardian/next of kin.

## Author contributions

KM, JR, and XL contributed to data acquisition, analysis, and interpretation. AL contributed to data analysis and manuscript revisions. DD and CL were responsible for study conception, funding, data interpretation, and manuscript revisions. All authors contributed to the article and approved the submitted version.
